# Desigualdades en el tiempo hasta el diagnóstico del síndrome de Down en Bolivia

**DOI:** 10.18294/sc.2024.4710

**Published:** 2024-03-19

**Authors:** Daniel Linares Terrazas, Beatriz Luna Barrón, Gonzalo Taboada López

**Affiliations:** 1 Médico. Auxiliar de investigación, Unidad de Citogenética, Instituto de Genética, Universidad Mayor de San Andrés, La Paz, Bolivia. dalinares1@umsa.bo Universidad Mayor de San Andrés Unidad de Citogenética Instituto de Genética Universidad Mayor de San Andrés La Paz Bolivia dalinares1@umsa.bo; 2 Médica. Docente investigadora, Instituto de Genética, Universidad Mayor de San Andrés, La Paz, Bolivia. bluna5@umsa.bo Universidad Mayor de San Andrés Instituto de Genética Universidad Mayor de San Andrés La Paz Bolivia bluna5@umsa.bo; 3 Médico. Docente investigador, Instituto de Genética, Universidad Mayor de San Andrés, La Paz, Bolivia. Universidad Mayor de San Andrés Instituto de Genética Universidad Mayor de San Andrés La Paz Bolivia

**Keywords:** Inequidades en Salud, Síndrome de Down, Equidad de Género, Diagnóstico Tardío, Bolivia, Health Inequities, Down Syndrome, Gender Equity, Delayed Diagnosis, Bolivia

## Abstract

El síndrome de Down es la condición genética más común y una causa principal de discapacidad intelectual. Las personas en áreas rurales, especialmente aquellas con discapacidades, a menudo enfrentan desigualdades en el acceso a la salud. A partir de los registros clínicos de pacientes con diagnóstico confirmado de síndrome de Down entre 2013 y 2022, por el Instituto de Genética de la Universidad Mayor de San Andrés, La Paz, Bolivia, se analizó, analizó el tiempo hasta el diagnóstico de 250 pacientes con síndrome de Down, mostró que los pacientes procedentes de áreas rurales con síndrome de Down tardan cinco meses en promedio en recibir un diagnóstico, comparado a los dos meses en zonas urbanas (p<0,001). No se encontraron diferencias significativas en el tiempo hasta el diagnostico según el sexo. Sin embargo, se evidenció una mayor proporción de varones provenientes de áreas rurales (p=0,03). Los hallazgos sugieren que los individuos de áreas rurales enfrentan dificultades para recibir el diagnóstico. Por otro lado, las mujeres quizás no sean llevadas a ciudades para un diagnóstico y tratamiento adecuado debido a sesgos de género en ciertas comunidades. Se subraya la importancia de mejorar el acceso a diagnósticos y tratamientos tempranos en áreas rurales.

## Abstract (en)

Down Syndrome is the most common genetic condition and a leading cause of intellectual disability. Individuals in rural areas, particularly those with disabilities, often face disparities in healthcare access. Analyzing clinical records of patients diagnosed with Down Syndrome between 2013 and 2022 by the Institute of Genetics at the Universidad Mayor de San Andrés in La Paz, Bolivia, this study examined the time to diagnosis for 250 patients with Down Syndrome. The findings revealed that patients from rural areas with Down Syndrome take an average of five months to receive a diagnosis, compared to two months in urban areas (p<0.001). No significant differences were found in the time to diagnosis based on gender. However, a higher proportion of males from rural areas was observed (p=0.03). The results suggest that individuals in rural areas face challenges in receiving a timely diagnosis. On the other hand, women may not be brought to cities for proper diagnosis and treatment due to gender biases in certain communities. The importance of improving access to early diagnosis and treatment in rural areas is emphasized.

## INTRODUCCIÓN

El síndrome de Down es un trastorno genético causado por la presencia de una copia extra del cromosoma 21, siendo la anomalía cromosómica más frecuente en todo el mundo y una de las principales causas genéticas de discapacidad intelectual^1^.

El término “intervención temprana” denota un conjunto de programas y recursos especializados brindados por profesionales de la salud a niños que presentan discapacidades específicas. Estas discapacidades abarcan diagnósticos tales como retraso del desarrollo, parálisis cerebral, trastorno del espectro autista, trastorno específico del lenguaje, síndrome de Down, entre otros. Estos programas se proporcionan tanto a los infantes afectados como a sus familias durante la etapa de la primera infancia, con el objetivo primordial de optimizar los resultados del desarrollo neuropsicológico^2^^,^^3^. Diversos estudios señalan que dicha intervención temprana tiene el potencial de mejorar de manera significativa los resultados y el progreso en individuos con síndrome de Down^4^^,^^5^. Por lo tanto, un diagnóstico temprano y oportuno y un enfoque adecuado se vuelven fundamentales para el manejo del síndrome de Down^6^. En consecuencia, brindar a estos individuos un apoyo temprano y adecuado, así como proporcionar a sus familias las herramientas necesarias, resulta esencial para potenciar su desarrollo y calidad de vida.

La desigualdad se caracteriza por la presencia de diferencias sistemáticas y potencialmente subsanables entre grupos poblacionales delineados por criterios socioeconómicos o geográficos^7^. Estas disparidades ejercen un notable impacto en la salud de los individuos. A modo ilustrativo, en España se ha documentado que los sujetos con un nivel educativo inferior exhiben una tasa de mortalidad que duplica a la de aquellos con una formación académica superior^8^. Adicionalmente, se ha detectado que los habitantes de zonas urbanas manifiestan un incremento más pronunciado en la esperanza de vida en comparación con sus contrapartes en áreas rurales, factor que amplifica la mencionada desigualdad^9^. En un ámbito conexo, las personas con discapacidades enfrentan una mayor propensión a ser objeto de discriminación y violencia^10^^,^^11^, situación que afecta de manera desproporcionada a las mujeres con discapacidad^12^^,^^13^.

En la región de Latinoamérica, se ha observado que las personas con discapacidad encuentran obstáculos al intentar acceder a los servicios de salud^14^. Esto es particularmente evidente en Bolivia donde, a pesar de algunos avances, persiste la falta de servicios gratuitos, aún contando con leyes que los respaldan^15^. La situación de las personas con discapacidad en Bolivia continúa marcada por la exclusión y la desigualdad, siendo frecuentemente objeto de discriminación^16^.

Al igual que afecta a personas con otras discapacidades, la desigualdad también impacta a quienes padecen síndrome de Down. Estas personas tienden a residir con mayor frecuencia en hogares de bajos ingresos, lo que, con el tiempo, incrementa sus probabilidades de caer en la pobreza y reduce sus oportunidades de superarla^17^. Además, persiste la discriminación racial en este contexto entre personas con síndrome de Down^18^. Aunque existe una carencia de evidencia que respalde la idea de retrasos en el diagnóstico del síndrome de Down en áreas rurales, la ubicación en comunidades rurales dificulta el acceso a un diagnóstico y atención pediátrica especializada^19^. 

Ante este contexto, el objetivo del presente estudio es analizar las desigualdades en el tiempo hasta el diagnóstico del síndrome de Down, según el lugar de residencia de los pacientes, ya sea en áreas rurales o urbanas, así como en función de su sexo. Adquirir una comprensión más profunda de cómo estos factores influyen en el diagnóstico de los pacientes con síndrome de Down sería fundamental para formular políticas públicas más efectivas destinadas a mejorar las condiciones de esta población.

## MATERIAL Y MÉTODOS

Se llevó a cabo un análisis retrospectivo de registros clínicos de pacientes con diagnóstico confirmado de síndrome de Down, mediante estudios citogenéticos en el Instituto de Genética de la Universidad Mayor de San Andrés, La Paz, Bolivia. Si bien el Instituto atiende principalmente a pacientes de La Paz debido a su reconocido prestigio, también recibe pacientes de diversas regiones de Bolivia. El Instituto de Genética de la Universidad Mayor de San Andrés es el principal centro de referencia para enfermedades genéticas de Bolivia, anualmente realiza de 100 a 150 estudios citogenéticos (cariotipos), recibe su financiamiento principalmente del sector público, mediante el presupuesto de la Universidad Mayor de San Andrés, que pertenece al sistema estatal de universidades de Bolivia. Los estudios realizados por el Instituto de Genética pueden ser solicitados por el médico tratante, por centros de salud o por hospitales pertenecientes al sistema de salud público o privado, por un costo mínimo. Históricamente, el Instituto de Genética ha sido el único centro estatal con la capacidad de realizar estudios de cariotipo no solo en la ciudad de La Paz, sino en Bolivia, desde las primeras décadas de su funcionamiento. Actualmente no es el único centro capaz de prestar este servicio, pero es el de mayor capacidad y experiencia en la temática, con 51 años de trabajo ininterrumpido. 

El análisis abarcó a todos los pacientes en los que se realizó un estudio de cariotipo en sangre por sospecha clínica de síndrome de Down, en el período comprendido entre 2013 y 2022, independientemente de la fecha de nacimiento de los casos. 

Los registros clínicos del Instituto de Genética guardan información sobre la anamnesis y examen físico de pacientes que acuden con solicitud de valoración por Genética, además de los resultados de los estudios genéticos realizados por la institución.

El procedimiento estándar realizado por el Instituto de Genética de la Universidad Mayor de San Andrés para la confirmación de los casos de síndrome de Down por trisomía del par 21 es mediante cariotipo, por medio de la toma de una muestra de 2 a 3 mililitros de sangre periférica para posterior siembra de la misma en medio específico, procediendo a la cosecha a las 72 horas de incubación a 37 °C, iniciando con colchicina para la detención en metafase e inducción de choque hipotónico con Cloruro de Potasio, para posteriormente lavar y fijar el pellet obtenido con solución Carnoy, y su visualización por microscopia simple en placas de portaobjetos envejecidas por 72 horas a 37 °C y bandeo G.

La principal variable de interés fue el intervalo de tiempo hasta el diagnóstico citogenético, considerado como el número de meses desde el nacimiento hasta la obtención de la muestra para el estudio citogenético, realizado por un especialista. Además, se recopilaron datos sobre el sexo y el lugar de residencia del paciente. 

Se considera área rural a las ciudades y localidades que están cubiertas por las redes rurales de salud, las cuales son definidas por los Servicios Departamentales de Salud (SEDES) de cada departamento de Bolivia. Según la Norma Nacional de Caracterización de Establecimientos de Salud de Primer Nivel, el término “área rural” se refiere a aquellas zonas con una población inferior a 30.000 habitantes y que están dentro del alcance de los centros de salud de primer nivel.

Para evaluar la normalidad de la distribución de los datos, se utilizó la prueba de Shapiro-Wilk. Debido a que los datos no siguieron una distribución normal, se presenta tanto la mediana y el rango intercuartílico como la media y la desviación estándar. Las variables continuas se analizaron mediante pruebas no paramétricas, específicamente la prueba de Mann-Whitney, mientras que para las variables categóricas se empleó el test exacto de Fisher. Se adoptó un valor de *p*<0,05 como criterio de significancia estadística. Todos los análisis se llevaron a cabo utilizando el software R, versión 4.2.2 (R Development Core Team, 2022).

Todos los datos fueron recopilados de manera retrospectiva en octubre de 2022 a partir de registros y se anonimizaron en el momento de la recolección con un número de estudio secuencial asignado para proteger las identidades y los registros personales de todos los pacientes. Este estudio fue aprobado por el comité de revisión del Instituto de Genética, el 7 de noviembre del 2023. Se utilizaron datos secundarios, por lo que no se requirió el consentimiento individual del paciente.

## RESULTADOS

De una muestra total de 250 casos confirmados de síndrome de Down mediante estudios citogenéticos, 61 casos (24,4%) provenían de áreas rurales, mientras que 189 (75,6%) pertenecían a zonas urbanas. Del total, 133 pacientes eran de sexo masculino (53,2%) y 117 de sexo femenino (46,8%).

**Figura 1:**
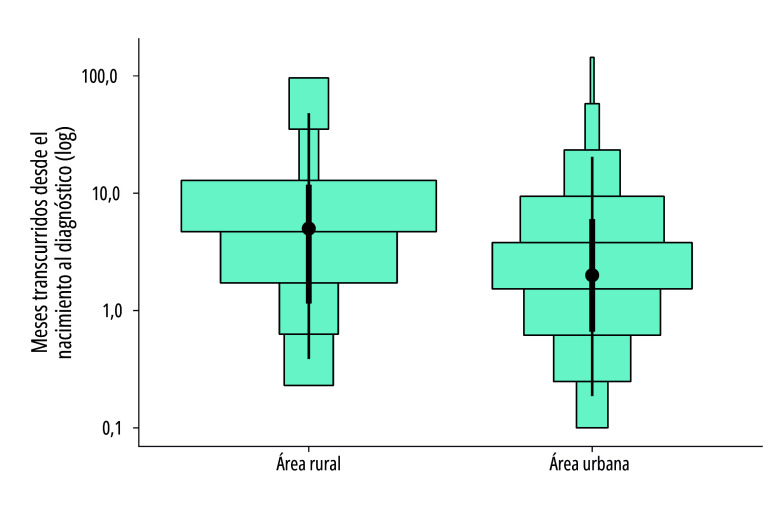
Comparación del tiempo hasta el diagnóstico de síndrome de Down, según área de residencia (rural y urbana) de los pacientes (n=250). Bolivia, 2013-2022

El análisis evidenció que, en áreas rurales, la mediana del tiempo hasta el diagnóstico fue de 5 meses de vida (rango intercuartílico = 7; media = 8,8, desviación estándar = 14,8), mientras que, en zonas urbanas, dicha mediana se situó en 2 meses (rango intercuartílico = 3; media = 4,6, desviación estándar = 11,7), como se ilustra en la Figura 1, lo que representa una diferencia significativa en el intervalo hasta el diagnóstico del síndrome de Down (*p*<0,001).

Fuente: Elaboración propia a partir de datos del Instituto de Genética de la Universidad Mayor de San Andrés, La Paz, Bolivia. Nota: Se combina un gráfico de cajas mediante las líneas verticales con un histograma de frecuencias (cajas de color celeste)

**Figura 2:**
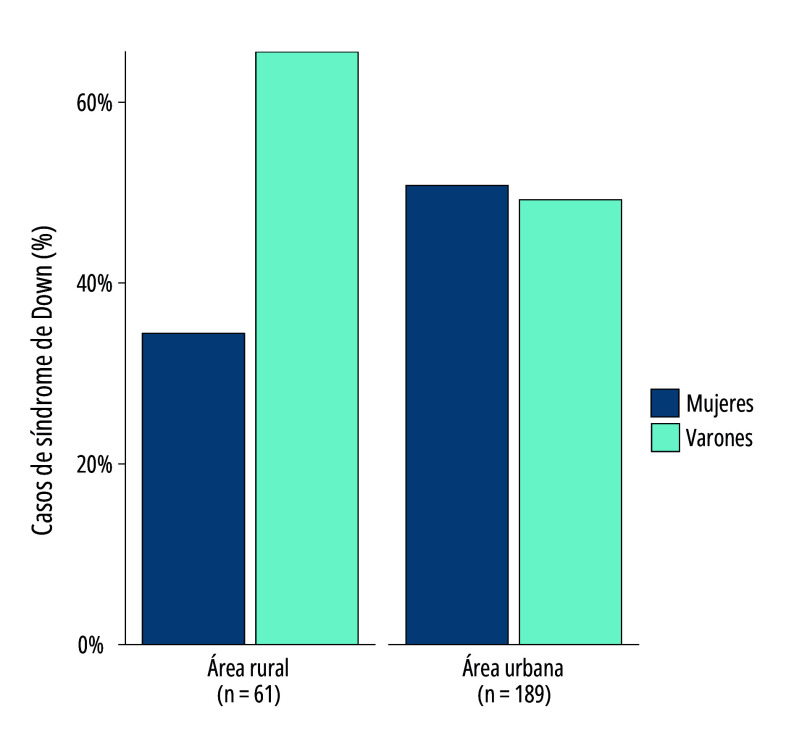
Distribución de pacientes diagnosticados con síndrome de Down (n=250), según sexo y área de residencia (rural y urbana). Bolivia, 2013-2022.

No se observaron diferencias significativas en el tiempo de diagnóstico según sexo (*p*=0,37). No obstante, la Figura 2 indica que, aunque en las áreas urbanas la proporción de pacientes varones diagnosticados (49,2%) y de mujeres (50,8%) es casi equitativa, en las áreas rurales predomina la proporción de pacientes masculinos diagnosticados (65,6%) en contraste con el 34,4% femenino, diferencia que resultó ser significativa (*p*=0,03).

Fuente: Elaboración propia a partir de datos del Instituto de Genética de la Universidad Mayor de San Andrés, La Paz, Bolivia.

## DISCUSIÓN

Los hallazgos del estudio subrayan una desigualdad significativa en el periodo requerido para diagnosticar el síndrome de Down entre los individuos residentes en zonas rurales en contraste con aquellos de áreas urbanas. En términos específicos, los infantes con síndrome de Down asentados en localidades rurales enfrentan un lapso diagnóstico considerablemente más extenso en relación con sus pares urbanos. Asimismo, se identificó una mayor proporción de varones derivados de regiones rurales, en contraposición a las áreas urbanas donde la proporción entre varones y mujeres referidos es aproximadamente equitativa. Esta tendencia sugiere la existencia de un posible sesgo de género en determinadas comunidades.

La identificación precoz del síndrome de Down resulta esencial para asegurar una intervención puntual y pertinente, la cual potencia el desarrollo y eleva la calidad de vida de los individuos afectados^6^. No obstante, un dilatado periodo para establecer el diagnóstico puede conllevar consecuencias adversas en el manejo de esta afección genética y sus comorbilidades concomitantes.

Diversos factores podrían estar influyendo en la disparidad observada respecto al intervalo diagnóstico entre las zonas rurales y urbanas. Notablemente, se subrayan elementos geográficos que generan variaciones en la disponibilidad y accesibilidad a servicios de salud y a expertos especializados^20^^,^^21^. Es plausible que las regiones rurales enfrenten obstáculos significativos al intentar acceder a servicios médicos y a profesionales con pericia en el diagnóstico del síndrome, prolongando así el periodo de diagnóstico^22^. La lejanía geográfica representa un factor crucial, dado que existen localidades considerablemente distantes de un establecimiento médico, lo cual obliga a las personas a organizar meticulosamente cada consulta médica, a menudo dejando de lado sus responsabilidades laborales. Adicionalmente, la insuficiencia de conocimiento o de información referente a los indicios iniciales del síndrome de Down entre los profesionales médicos y progenitores en comunidades rurales podría tener un papel determinante en la dilatación diagnóstica.

En la actualidad, se evidencia una carencia de investigaciones que aborden la influencia de diversos factores en los tiempos de diagnóstico en pacientes con síndrome de Down en la región latinoamericana. No obstante, se ha constatado que, en Bolivia, y en la mayoría de los países latinoamericanos, las personas con discapacidad experimentan significativas barreras al intentar acceder a los servicios de salud^15^^,^^16^.

Hay que tener en cuenta diversos elementos adicionales, entre los cuales figuran factores socioculturales como el estatus socioeconómico, el grado de alfabetización y la pertenencia étnica. Dichos factores pueden estar influenciando las desigualdades observadas entre pacientes con síndrome de Down asentados en regiones rurales y urbanas. Es ampliamente reconocido que variables sociodemográficas, como el estatus socioeconómico y el nivel educativo de los padres, ejercen una notable influencia en el desarrollo neurolingüístico de los sujetos con síndrome de Down^23^. Paralelamente, en ciertas poblaciones, se ha evidenciado la existencia de desigualdades de carácter racial en relación con la esperanza de vida de las personas diagnosticadas con síndrome de Down. En particular, aquellos afectados que provienen de entornos socialmente desfavorecidos tienden a manifestar una expectativa de vida reducida^24^^,^^25^^,^^26^.

Adicionalmente, hay factores intrínsecos al individuo que deben ser considerados. A pesar de no haber identificado diferencias significativas en el tiempo hasta el diagnóstico basándonos en el sexo, se evidenció una variación en la distribución de pacientes por sexo en función de su área de residencia. En las zonas urbanas, la proporción de varones y mujeres diagnosticados es equiparable; sin embargo, los pacientes derivados de áreas rurales son predominantemente varones. Este dato sugiere que las niñas con síndrome de Down en zonas rurales enfrentan mayores obstáculos para obtener un diagnóstico y, consecuentemente, un tratamiento adecuado. Es un hecho reconocido que las mujeres con discapacidades a menudo enfrentan barreras en el acceso a la atención sanitaria^12^^,^^27^. Así, las dificultades para obtener atención médica podrían intensificarse para las mujeres con discapacidades procedentes de áreas rurales, lo que podría justificar la elevada proporción de varones diagnosticados en estas regiones. Por otro lado, los pacientes con síndrome de Down no están exentos de la discriminación racial^18^, planteando desigualdades aún mayores a las observadas en la población general^28^. Sin embargo, si bien este fenómeno es poco estudiado en Latinoamérica, ha cobrado gran relevancia, dado que las poblaciones rurales son más susceptibles a distintas formas de discriminación^29^. 

Los factores técnicos en un laboratorio resultan esenciales, especialmente si se toma en cuenta el lapso entre el nacimiento y la obtención de muestras para el análisis del cariotipo. Este proceso implica una espera adicional de entre 10 y 30 días, atribuible al cultivo celular y otros procedimientos específicos al tipo de estudio. Se admiten únicamente las muestras de pacientes que no se encuentren bajo tratamiento antibiótico, que no hayan recibido transfusiones recientemente y que no presenten ictericia^30^. En determinados casos, puede ser necesario obtener una segunda muestra para lograr un diagnóstico certero. Este escenario cobra especial importancia para individuos con síndrome de Down residentes en zonas rurales: al encontrarse alejados de los centros de atención, deben interrumpir sus actividades cotidianas para asistir a consultas o recolectar las muestras. Esta situación repercute negativamente en su estabilidad económica, profundizando la brecha entre las áreas rurales y urbanas.

Es crucial subrayar que la desigualdad y el retraso en el diagnóstico afectan no solo a las personas con síndrome de Down, sino también a sus familias. Estas últimas requieren información, apoyo y recursos para enfrentar la condición de sus seres queridos^31^. La carencia de acceso a un asesoramiento genético adecuado restringe la obtención de información sobre la enfermedad, obstaculizando la toma de decisiones informadas al respecto.

La convergencia de estos factores resulta en un retraso en el diagnóstico, del que se desprenden tres implicaciones esenciales. Primero, hay una repercusión en el tiempo que requiere la familia para asimilar la enfermedad, lo que obstaculiza la adaptación al nuevo escenario y complica la aceptación de la patología, limitando así un ajuste efectivo a la vida con tal condición^32^^,^^33^. En segundo lugar, se manifiesta una insuficiencia en el acceso a la estimulación temprana, lo que podría limitar el desarrollo neurocognitivo a largo plazo del paciente^3^. Finalmente, el no reconocimiento de comorbilidades ligadas al síndrome Down, como las cardiopatías congénitas o el hipotiroidismo -que cuentan con tratamientos que optimizan significativamente el pronóstico-, entraña un peligro de complicaciones permanentes en fases subsiguientes, afectando directamente la calidad de vida del sujeto^34^^,^^35^^,^^36^^,^^37^^,^^38^. 

El presente estudio no está exento de limitaciones, la realización del estudio en un instituto genético especializado puede limitar la generalización a contextos sanitarios más amplios. Además, el diseño retrospectivo plantea intrínsecamente dificultades para establecer relaciones causales y puede ser susceptible de sesgo de recuerdo. La falta de información socioeconómica detallada puede dificultar un análisis exhaustivo de los posibles factores de confusión. Por lo cual, se requieren más investigaciones para confirmar los hallazgos.

Para enfrentar la disparidad en el tiempo de diagnóstico, es imperativo implementar intervenciones a nivel de políticas sanitarias y educativas. Es fundamental instaurar programas de formación y sensibilización dirigidos a los profesionales sanitarios en regiones rurales, de modo que estén capacitados para reconocer precozmente los indicativos del síndrome de Down y puedan canalizar a los pacientes hacia especialistas de manera adecuada, a través de sistemas eficientes de referencia y contrarreferencia. Asimismo, es esencial potenciar los servicios de salud y educación en áreas rurales para optimizar el acceso a la atención médica y a herramientas especializadas. Además, es crucial enfrentar el sesgo de género presente en ciertas comunidades que podría obstaculizar el diagnóstico temprano de niñas con síndrome Down, sensibilizando a las comunidades sobre el perjuicio que representa la falta de atención médica adecuada.
